# Occupational Health Aspects with Special Focus on Physiological Differences between Office and Metalworkers

**DOI:** 10.3390/antiox11040633

**Published:** 2022-03-25

**Authors:** Franz Tatzber, Sieglinde Zelzer, Barbara Obermayer-Pietsch, Stefan Rinnerhofer, Michael Kundi, Gerhard Cvirn, Georg Wultsch, Markus Herrmann, Harald Mangge, Tobias Niedrist, Willibald Wonisch

**Affiliations:** 1 Otto Loewi Research Center, Division of Immunology and Pathophysiology, Medical University of Graz, Heinrichstraße 31a, 8010 Graz, Austria; franz.tatzber@medunigraz.at; 2 Clinical Institute of Medical and Chemical Laboratory Diagnostics, Medical University of Graz, Auenbruggerplatz 29, 8036 Graz, Austria; sieglinde.zelzer@medunigraz.at (S.Z.); markus.herrmann@medunigraz.at (M.H.); harald.mangge@medunigraz.at (H.M.); tobias.niedrist@medunigraz.at (T.N.); 3 Endocrinology Lab Platform, Department of Internal Medicine, Division of Endocrinology and Diabetology, Medical University of Graz, Auenbruggerplatz 15, 8036 Graz, Austria; barbara.obermayer@medunigraz.at; 4 Exercise Physiology, Training and Training Therapy Research Group, Institute of Sports Science, University of Graz, Mozartgasse 14, 8010 Graz, Austria; stefan.rinnerhofer@sportunion-steiermark.at; 5 Center for Public Health, Department of Environmental Health, Medical University of Vienna, Kinderspitalgasse 15, 1090 Vienna, Austria; michael.kundi@meduniwien.ac.at; 6 Otto Loewi Research Center, Division of Physiological Chemistry, Medical University of Graz, Neue Stiftingtalstraße 6 HBK M1/D3, 8010 Graz, Austria; gerhard.cvirn@medunigraz.at; 7 Arbeitsmedizinisches Institut Graz, Herrgottwiesgasse 149, 8055 Graz, Austria; office@georg-wultsch.at

**Keywords:** oxidative stress, sex-specific differences, taxing work, exhaustion, white-collar worker, blue-collar worker

## Abstract

Physical workload adversely impacts inflammation, oxidative stress and mood in heavy workers. We compared these risk parameters between metalworkers (*n* = 20) and office workers (*n =* 30), including gender differences. Blood samples were analyzed with thirty parameters to overview endocrinology, inflammation, and psychological and oxidative stress. Despite an adequate antioxidative supply, oxidative stress occurred in metalworkers, as indicated by significantly increased peroxide and homocysteine (Hcy) levels. Moreover, increased concentrations were observed in this group regarding psychological stress and diet-related parameters. Sex-specific differences were determined for physical dimensions, dehydroepiandrosterone sulfate (DHEAS), Hcy, uric acid, triglycerides, osmolality, anti-Mullerian hormone (AMH) and testosterone. Age-associated differences were observed for DHEAS, glycosylated hemoglobin, adrenaline, AMH and testosterone. In male office workers, the body mass index was associated with increased LDL-HDL, cholesterol-HDL and homeostatic model assessment of insulin resistance (HOMA-IR). In conclusion, these results indicate increased oxidative stress and psychological stress in heavy workers independently of adequate antioxidant sustenance. The sedentary occupation of office workers, in turn, favored diseases of affluence. This might be particularly relevant for long-term occupied persons and older workers due to a hormonal shift coming along, given the risk for oxidative stress-related diseases such as cardiovascular disease, particularly in the case of males, based on their lifestyle habits.

## 1. Introduction

Various influences are decisive in the workplace, contributing to the impairment of work performance, i.e., easy or heavy work, environmental conditions such as fumes or dust, diet and imbibition, and travel time to the worksite. Manual work is associated with a diminished anti-oxidative capacity accompanied by increased oxidative stress and even DNA damage, as shown in workers of a copper processing company [1]. This might be connected with an intense exercise-associated increase in homocysteine (Hcy), independently of the vitamin status or type of exercise as a result of increased creatinine concentrations related to changes in the renal function [2]. Low-grade inflammation was shown in a quarter of workers with significant interactions of long work durations and shift work as an excess risk in middle-aged men [3]. Inflammation and oxidative stress increased the risk for cardiovascular toxicity in welding workers compared to office workers due to metal fumes [4]. One cause might be a heightened risk of diabetes in metalworkers compared to office workers, as shown previously [5]. Multimorbidity affects workers’ health through detrimental effects to quality of life and work productivity, an increase in the sickness absence rate and earlier retirement [6]. In office workers, a prevalence of 35.9% for metabolic syndrome was associated with lack of leisure time, physical activity and low intake of fruits [7]. Sleep problems associated with a long commute time was shown in workers for both sexes in a large cross-sectional study [8]. Biomarkers for oxidative stress and antioxidants are among the most important predictors of workload and exhaustion [9]. As oxidative stress is correlated with inflammation [10], high-intensity exercise also increases high-sensitive C-reactive protein (hsCRP) [11]. Sex-specific differences in key-markers of inflammation and immune activation have an impact on stress-related diseases and morbidity in further consequence [12]. Gender-specific differences were reported in a large-scale cross-sectional study [13] in which most diet-related parameters, such as body-mass index (BMI), uric acid or bilirubin, were elevated in males, due to an improved health consciousness and regimen in females, as this was concluded by the authors. Interestingly, the peroxide level was increased three-fold in females. One reason for that might be the use of oral contraceptives that increase oxidative stress and CRP levels compared to naturally cycling women, which might have implications even on workload [14]. In addition, metalworkers were associated with adverse neurobehavioral effects right up to the disruption of the endocrine system, such as low testosterone and follicle-stimulating hormone levels upon high-levels of manganese [15].

In the present study, we investigated the workload in office workers versus metalworkers, emphasizing oxidative and psychological stress, inflammation, and endocrinological and hormonal aspects, including sexual differences. We hypothesized that heavy workers are associated with increased oxidative and psychological stress and may be prone to an enhanced risk for oxidative-stress-related diseases, such as cardiovascular disease, particularly with the increasing length of employment and age.

## 2. Materials and Methods

### 2.1. Study Population

In this cross-sectional study, we enrolled a total of 41 male and 9 female volunteers at their worksite. Thereof, 30 subjects were office employees (4 females) with a mean age of 42.5 (39.9–45.0) years and 20 workers (5 females) from a metalworking company with a mean age of 35.3 (32.1–38.5) years. Exclusion criteria were infections, chronic diseases and certified reduced work capacity due to illness. The study was conducted in accordance with the Helsinki Declaration and was approved by the ethics committee of the Medical University of Graz, Austria (EK number 26-488 ex 13/14). All participants provided written informed consent. The study was performed between 4 October 2018 (FPI) and 18 November 2019 (LPO).

### 2.2. Blood Sampling

Blood was drawn from an antecubital vein from the seated subject between 6:00 am and 8:00 am, before an eight-hour work shift. Samples were transferred immediately on ice to the laboratory within two hours. After that, they were centrifuged and stored at −80 °C until analysis within 6 to 18 months.

### 2.3. Laboratory Analysis—Routine Procedures and Cutting-Edge Biomarkers

Creatinine, glucose, uric acid, high-sensitive C-reactive protein (hsCRP), total cholesterol, HDL-cholesterol, and triglycerides were measured with a COBAS 8000 analyzer (Roche Diagnostics GmbH, Mannheim, Germany). LDL-cholesterol was estimated using the Friedewald equation. Homocysteine (Hcy) was determined on an Architect analyzer (Abbott Laboratories, Sligo, Ireland).

Adrenocorticotropic hormone (ACTH) was determined using a solid-phase two-site sequential chemiluminescent immunometric assay (Immulite 2000, Siemens, Erlangen, Germany).

Cortisol—analyses were performed by solid-phase two-site sequential chemiluminescent immunometric assay (Advia Centaur, Siemens, Erlangen, Germany).

Dehydroepiandrosterone sulfate (DHEAS) was analyzed by ELISA (LDN GmbH, Nordhorn, Germany).

Glycosylated haemoglobin (HbA1c) was analyzed with a reversed-phase cation exchange chromatography dual-wavelength method on a Menarini HA 8180T analyzer (A. Menarini Diagnostics, Florence, Italy). The measurement range of the HbA1c concentration was between 9 and 195 mmol/mol (3–20%).

Homeostatic model assessment of insulin resistance (HOMA-IR) was calculated by the equation:

Fasting Insulin Value (µU/mL) × Fasting Blood Glucose (mg/dL)/405.

Copeptin was measured by an immune-luminometric assay by Brahms, Germany.

Osmolality (OL) of body fluid was measured with the ultra-cooling method using the automatic osmometer OSMO STATION OM-6060 (ARKRAY Inc., Kyoto, Japan).

Adrenaline, noradrenaline, and dopamine were determined by radioimmunoassay from DRG Instruments GmbH, Marburg, Germany.

Anti-Mullerian hormone (AMH) was analyzed by a chemoluminescence immunoassay (Immunotech by Beckmann Coulter Inc., Marseille, France).

Androstenedione was measured by a solid-phase two-site sequential chemiluminescence immunometric assay (Advia Centaur, Siemens, Erlangen, Germany).

Follicle-stimulating hormone (FSH) was analyzed by a chemoluminescence immunoassay (Beckmann Coulter Inc., Nyon, Switzerland).

Free testosterone was measured by radioimmunoassay from Immunotech (Beckmann Coulter, Prague, Czech Republic).

Total testosterone was analyzed by a solid-phase two-site sequential chemiluminescence immunometric assay (Advia Centaur, Siemens, Erlangen, Germany).

Total Antioxidant Capacity (TAC) was determined with a colorimetric method supplied by LDN (Labor Diagnostic Nord, Nordhorn, Germany). Antioxidants present in the samples inhibit the attack of free radicals, which are initiated through a peroxide/peroxidase start-reaction. Therefore, they reduce the colorimetric signal of the substrate tetramethylbenzidine. Briefly, 25 µL sample volume was incubated with 100 µL reagent A, consisting of 30% hydrogen peroxide and citrate-substrate buffer in a proportion of 1:1000 and 50 µL reagent B, consisting of horseradish peroxidase (25 mU), 3,5,3′5′-Tetramethylbenzidine (TMB), and a citrate-substrate buffer in a proportion of 1:10:1000, in uncoated microtiter plates for 20 min at four °C. The stop solution’s addition finally caused a color change from blue to yellow, and the absorbance was detected at 450 nm (reference wavelength 620 nm). Trolox equivalents (mmol/L) were used for the quantification of samples. All analyses were done in duplicate, and the results are expressed in mmol/L as Trolox equivalents.

Total polyphenols (PPm) were determined with a previously published high-throughput method [16] supplied by Omnignostica Ltd. (Höflein/Danube, Austria). This method is a modified version of the classical Folin–Ciocalteu reaction, i.e., polyphenols with transition metals, to obtain a dark-colored complex within the incubation period of at least 2 h. Serial dilutions of gallic acid were used to quantify sample polyphenols at a wavelength of 766 nm. All analyses were done in duplicate, and results were expressed in mmol/L as gallic acid equivalents.

Total Peroxides (TOC) and Endogenous Peroxidase-Activity (EPA)—were determined with colorimetric assays according to the method of Tatzber et al. [17] and were supplied by LDN (Labor Diagnostic Nord, Nordhorn, Germany). The assays are based on the reaction between peroxides and horseradish peroxidase in the presence of the substrate TMB. After the addition of stop-solution, the color changes to yellow and is measured at a wavelength of 450 nm (ref. 620 nm). Quantification was done by a linear standard curve of each and presented as µmol/L in the case of TOC and U/L for EPA.

### 2.4. Statistics

No formal sample-size determination was done since the groups of metal and office workers available to us had very limited sizes. However, we performed a power analysis that revealed a power of 80% to detect a difference between groups of 0.84 standard deviations at a test-wise significance level of 5%. Since a large number of tests have been performed, there is a risk of alpha inflation, and therefore significant findings should be considered exploratory.

Metric variables were summarized as mean and 95% confidence intervals (95% CI). Before further analysis, all dependent variables were subjected to distribution analysis. Most laboratory variables showed the best fit of a log-normal distribution and were, therefore, log-transformed before further analysis. All dependent variables were analyzed by the General Linear Model with gender, age, and body mass index (BMI) as covariates and occupational group (metalworkers vs. office workers) as the main independent variable of interest. Homogeneity of variances was assessed by Brown–Forsythe tests and normality of residuals by Kolmogorov–Smirnov tests with Lilliefors’ corrected *p*-values. In case of a significant effect of age and/or BMI, correlations between the respective dependent variable and age and/or BMI were computed separately for the combination of gender and occupational group. Analyses were done by Statistica 10.0 (StatSoft, Tulsa, OK, USA). *p*-values below 0.05 were considered significant. No correction for multiple endpoints was applied.

## 3. Results

### 3.1. Group-Specific Differences

Several biomarkers regarding oxidative stress, diet and mood were significantly different between metalworkers and office workers.

Peroxides (TOC) are generated as early breakdown products mediated through the attack of reactive oxygen species on fatty acids in a chain-reaction, designated as lipid peroxidation. The TOC was significantly increased in metalworkers (MW) (*p =* 0.001), i.e., 225 µmol/L (=mean; 189–262 µmol/L; 95% CI) compared to office workers (OW) 141 µmol/L (=mean; 112–170 µmol/L; 95% CI).

Homocysteine was significantly increased as well, with a mean of 15.9 µmol/L (14.3–17.8 µmol/L; 95% CI) in metalworkers versus 11.6 µmol/L (=mean; 10.7–12.7 µmol/L; 95% CI; *p <* 0.001) in office workers. There was also a trend for augmented inflammation in metalworkers with a mean hsCRP level of 1.75 mg/L (1.09–2.82 mg/L; 95% CI) compared to 0.99 mg/L (=mean; 0.68–1.44 mg/L; 95% CI) in office workers, albeit not significant (*p =* 0.080).

Diet-related markers as indicators of a different diet were significantly increased in metalworkers, i.e., triglycerides (MW-mean = 126 mg/dL; 105–153 md/dL; 95% CI vs. OW-mean = 82.76 mg/dL; 71.4–96 mg/dL; 95% CI; *p =* 0.001), HbA1c (MW-mean = 32.9 mmol/mol; 31.8–34.00 mmol/mol; 95% CI vs. OW-mean = 31.1 mmol/mol; 30.2–31.9 mmol/mol; 95% CI; *p =* 0.016), HOMA-IR (MW-mean = 1.51; 1.17–1.95; 95% CI vs. OW-mean = 0.96; 0.78–1.17; 95% CI; *p =* 0.011), osmolality (MW-mean = 289 mosmol/kg; 287–290 mosmol/kg; 95% CI vs. OW-mean = 285 mosmol/kg; 283–286 mosmol/kg; 95% CI; *p <* 0.001).

Except for age (OW-mean = 42.5 years; 39.9–45.0 years; 95% CI vs. MW-mean = 35.3 years; 32.1–38.5 years; 95% CI; *p <* 0.001) and adrenaline, which was increased in OW (mean = 3.08 µg/g creatinine; 2.41–3.93 µg/g creatinine; 95% CI vs. MW-mean 1.58 µg/g creatinine; 1.10–2.27 µg/g creatinine; 95% CI; *p =* 0.004), several psychological stress and mood parameters were also increased in metalworkers, i.e., DHEAS (mean = 342 µg/dL; 292–402 µg/dL; 95% CI vs. OW-mean = 263 µg/dL; 232–299 µg/dL; 95% CI; *p =* 0.017), ACTH (MW-mean = 31.2 pg/mL; 24.6–39.7 pg/mL; 95% CI vs. OW-mean = 19.6 pg/mL; 16.3–23.7 pg/mL; 95% CI; *p =* 0.005), cortisol (MW-mean = 143 ng/mL; 124–165 ng/mL; 95% CI vs. OW-mean = 94.9 ng/mL; 84.9–106.2 ng/mL; 95% CI; *p <* 0.001) and androstenedione (MW-mean = 2.44 ng/mL; 2.05–2.89 ng/mL; 95% CI vs. OW-mean = 1.88 ng/mL; 1.64–2.15 ng/mL; 95% CI; *p =* 0.027).

### 3.2. Sex-Specific Differences

As expected, height (*p <* 0.001), weight (*p <* 0.001) and even age (*p =* 0.008) were increased in males compared to females.

In addition to this, several parameters were significantly increased in males. Data are presented as mean and 95% confidence interval (CI):

DHEAS (*p =* 0.04): male OW 274 µg/dL (239–315 µg/dL) vs. female OW 224 µg/dL (159–313 µg/dL) and in male MW 364 µg/dL (307–432 µg/dL) vs. female MW 252 µg/dL (174–363 µg/dL).

Uric acid (*p <* 0.001): male OW 5.76 mg/dL (5.42–6.09 mg/dL) vs. female OW 4.87 mg/dL (4.04–5.70 mg/dL)–male MW 5.75 mg/dL (5.33–6.17 mg/dL) vs. female MW 4.26 mg/dL (3.36–5.17 mg/dL).

Homocysteine (*p =* 0.004): male OW 12.37 µmol/L (11.30–13.53 µmol/L) vs. female OW 8.04 µmol/L (6.45–10.02 µmol/L)–male MW 16.20 µmol/L (14.50–18.09 µmol/L) vs. female MW 14.93 µmol/L (11.76–18.95 µmol/L).

Triglycerides (*p =* 0.01): male OW 87.3 mg/dL (74.4–102.6 mg/dL) vs. female OW 66.9 mg/dL (45.1–99.1 mg/dL)–male MW 139 mg/dL (114–169 mg/dL) vs. female MW 78.3 mg/dL (51.1–120 mg/dL).

Osmolality (*p =* 0.001): male OW 285 mosmol/kg (284–287 mosmol/kg) vs. female OW 282 mosmol/kg (278–285 mosmol/kg)–male MW 290 mosmol/kg (288–291 mosmol/kg) vs. female MW 284 mosmol/kg (280–288 mosmol/kg).

AMH (*p <* 0.001): male OW 4.53 ng/mL (3.64–5.65 ng/mL) vs. female OW 0.67 ng/mL (0.12–3.90 ng/mL)–male MW 6.20 ng/mL (4.66–8.24 ng/mL) vs. female MW 1.96 ng/mL (0.30–12.96 ng/mL).

Free testosterone (*p <* 0.001): male OW 8.45 pg/mL (7.62–9.36 pg/mL) vs. female OW 0.90 pg/mL (0.44–1.84 pg/mL)–male MW 9.76 pg/mL (8.47–11.25 pg/mL) vs. female MW 1.34 pg/mL (0.67–2.67 pg/mL).

Total testosterone (*p <* 0.001): male OW 3.45 ng/mL (2.56–4.65 ng/mL) vs. female OW 0.12 ng/mL (0.06–0.24 ng/mL)–male MW 4.77 ng/mL (4.09–5.55 ng/mL) vs. female MW 0.19 ng/mL (0.11–0.33 ng/mL).

### 3.3. Age-Specific Differences

Age-related differences were observed for DHEAS and AMH, which decreased significantly (*p =* 0.006) in male office workers. In addition, free-testosterone decreased significantly in males of both groups, i.e., in OW (*p =* 0.001) and MW (*p =* 0.044), whereas total testosterone decreased exclusively in MW (*p =* 0.032). Furthermore, HbA1c increased with age in male metalworkers (*p =* 0.05) in contrast to adrenaline, which was associated with a significant decrease in this group (*p =* 0.001).

### 3.4. BMI-Specific Differences

In relation to BMI a significant increase in the LDL/HDL ratio (*p =* 0.016), cholesterol/HDL ratio (*p =* 0.026) and HOMA-IR (*p =* 0.006) was observed in male office workers. In male metalworkers, there was a trend to increased FSH (*p =* 0.052). For details, see Table 1 and Table 2.

### 3.5. Spearman Correlations

Triglycerides were significantly correlated to uric acid (OW: *p =* 0.008; MW: *p =* 0.04) and HOMA-IR (OW: *p =* 0.001; MW: *p =* 0.002).

TAC was correlated to EPA (OW: *p =* 0.049; MW: *p <* 0.001) in both groups.

## 4. Discussion

Oxidative stress was proved in our study in metalworkers despite an excellent antioxidative sustenance, as indicated by significantly increased peroxide levels (225 µmol/L) compared to office workers (141 µmol/L; *p =* 0.001), which were even above the standard range (upper limit: 150–200 µmol/L). Peroxides are among the most sensitive parameters for oxidative stress, as reported previously [18,19]. Peroxide levels in metalworkers were distinctly worse compared to another group of heavy workers [10] independent of adequate antioxidant sustenance. A reason for this might be the ambient temperature [20] and environmental conditions, such as smoke or metal residues that are harmful to health [1,4]. This is consistent with a recent publication that found increased oxidative stress in workers exposed to different types of nanomaterials compared to office workers [21,22]. Metal exposure and duration was also correlated to arterial hypertension as a result of increased oxidative stress [23]. Exercise capacity, muscle oxygenation and the psychological impact are negatively associated with higher C-reactive protein levels, indicating poorer quality of life for subjects with higher CRP concentrations [24]. High levels of effort, over-commitment and effort–reward imbalance at work increase CRP levels significantly in both sexes and link job stress and stress-related diseases such as atherosclerosis [25,26]. There was also a trend of increased pro-inflammatory processes in metalworkers, as indicated by elevated hsCRP concentrations (1.75 mg/L vs. 1.0 mg/L in office workers), in the present study, albeit not significant (*p =* 0.08). There is also growing evidence that the gut microbial function is different between active and sedentary subjects. The microbial diversity was correlated with physical activity, as indicated by muscle strength and calf circumference [27]. This seems to be dependent on body composition, body fat, and exercise intensity, as there are also reports of detrimental consequences of exercise on exhaustion for the microbiome [28]. Although the research on work-related exposures is currently sparse, it should be emphasized that entire microbial communities may shift towards a pro-inflammatory state in non-communicable diseases such as obesity or diabetes [29]. Moreover, environmental exposure to chemicals or metals can also change the human microbiome to dysbiosis. In an animal study, heavy metals accumulated primarily in the kidneys of *Cyprinus carpio* and induced the generation of reactive oxygen species with elevated malondialdehyde and superoxide dismutase levels over several weeks [30]. Therefore, this complex item should be investigated in further studies.

Homocysteine levels above 15 µmol/L are defined as homocysteinemia, a risk factor for cardiovascular disease. Besides an excess of total peroxides, metalworkers also indicated an excess of homocysteine, i.e., 15.9 µmol/L (14.3–17.8 µmol/L; 95% CI) in comparison to office workers with 11.6 µmol/L (=mean; 10.7–12.7 µmol/L; 95% CI; *p* < 0.001) that is conterminous with the definition of homocysteinemia, especially in males. Homocysteine seems to be a two-sided sword because it has antioxidative properties besides the negative impact on cardiovascular diseases, e.g., the efficient stoichiometrically reduction of dehydroascorbic acid (DHA) to ascorbic acid (AA) at low concentrations as 10 µmol/L. In this respect, it was even more effective than reduced glutathione or cysteine. Moreover, the oxidation of homocysteine prevents a decrease in DNA synthesis compared to reduced homocysteine in human umbilical vein endothelial cells. Thus, this bivalent molecule indicates excess oxidative stress and regenerates ascorbic acid as a countermeasure [31]. The regeneration of DHA to AA is vital to protect against low-density lipoprotein (LDL) oxidation by Iron (III) that emerges during exhausting exercise or inflammation, while copper chelation by Hcy is very effective against LDL-oxidation by copper (II) [32]. The fact that homocysteine concentrations were significantly decreased in multivitamin users is probably the result of the antioxidative impact on free radicals that prevent a homocysteine increase. This underlines the biphasic role of this molecule as a stress indicator on the one hand and as second-line prevention on the other hand [33]. This is complementary to a previous study, where an oral supplementation of folic acid in coronary artery disease patients revealed a two-edged pattern, dependent on the homocysteine reduction. Peak reactivity of resistance vessels improved significantly in subjects with a homocysteine reduction of greater than 2 µM, whereas there was no effect on the antioxidant status. In subjects below this cut-off, the peak reactivity of resistance vessels was unaltered, but the antioxidant status increased significantly [34]. The difference in homocysteine levels between sexes was attributed to the requirement for labile methyl groups, whose turnover is linked to the synthesis of creatine/creatinine, which is related to a higher muscle mass in males sex hormones and creatinine values. In females, the homocysteine concentration depends on the menstrual cycle and is closely associated with estrogen status [35,36].

As a factor of physical and psychological stress, the ACTH level was significantly increased (*p =* 0.005) in workers (31.2 pg/mL) compared to office workers (19.6 pg/mL), which corresponds to a previous report in the case of slaughterhouse workers [10]. Furthermore, increased psychological stress associated with bad mood was observed in metalworkers by elevated levels of DHEAS, androstenedione and cortisol. It is noteworthy that adrenaline was significantly increased in office workers. This might be a distinct part of psychological stress, which corresponds to stress-related diseases, particularly among managers [37]. Furthermore, increased biomarkers for imbibition and diet were observed in metalworkers, e.g., osmolality, triglycerides, HbA1c and HOMA-IR. In this respect, it was reported that HOMA-IR levels were associated with poorer general health and adverse changes across several biochemical markers in normal individuals as well as in prediabetic and diabetic subjects in a large population-based sample from the “National Health and Nutrition Examination Survey” [38]. HOMA-IR is positively associated with high dietary acid load and serum calcium concentrations and inversely associated with serum phosphate concentrations and dietary as well as serum magnesium and HbA1c, which was investigated in a Japanese population of more than 1700 workers [39,40,41]. Moreover, HOMA-IR was shown to correlate with oxidized LDL and hsCRP in shift workers, indicating a rise in insulin resistance and oxidative stress and an increased risk of systemic inflammation [42,43]. In addition, the chronic stress marker cortisol was found to be an independent predictor of HOMA-IR and the association with insulin resistance in both sexes [44]. As a countermeasure, physical exercise effectively decreased glucose and HOMA-IR in young men and women as a strategy to improve glycemic control [45].

Sex-specific differences are primarily related to imbibition, diet and health consciousness between males and females. Especially in female office workers, an insufficient intake of fluids was observed that was significantly correlated to serum osmolality. Considerably lower lipid levels and uric acid concentrations in females were shown in a large cross-sectional study [13]. This corresponds to our results indicating a worse regimen in males associated with increased homocysteine concentration, besides distinct hormonal differences. It should be pointed out that, other than in the aforementioned report, there were no significant differences of total peroxide levels between genders in the present study, but significant differences occurred between groups.

Age-related differences were observed for DHEAS and AMH, which significantly decreased in office workers (*p =* 0.006). A recent report showed that regular physical activity was associated with increased DHEAS levels, which is particularly beneficial for well-being in old age [46]. Due to the lower physical load in office workers, the decrease in DHEAS is consistent with age. The decrease in serum anti-Mullerian hormone with age corresponds to other reports [47,48], prevalently in sedentary subjects as office workers because AMH levels are significantly higher in exercised subjects [49]. Moreover, an inverse correlation exists between AMH and CRP in men [50], which is associated with increased mortality [51]. Total testosterone in metalworkers and free testosterone in both groups were indirectly related to age, which is coincident with a report of Kanabar et al. [52] indicating a steep decline around the age of 30 to 40 years. These authors also found an inverse relationship between testosterone and HbA1c, which is consistent with the increase in this parameter in metalworkers of the present study, indicating a predecessor of diabetes and metabolic syndrome. Decreased adrenaline levels in metalworkers are advantageous because physical training, i.e., a combination of resistance and aerobic exercise in men, was associated with improving body composition cardiometabolic risk factors and physical performance [53].

In the case of office workers, eating behavior and sedentary occupation were correlated to BMI, i.e., increased LDL–HDL ratios, cholesterol/HDL ratios and HOMA-IR. This might be correlated to inflammatory mediators and increased carotid intima-media thickness indicating subclinical atherosclerosis [54,55].

The strength of this work is the comprehensive determination of routine and innovative biomarkers to establish hormonal status, lipid and glucose metabolism, kidney, psychological and oxidative stress factors. Thus, significant differences and correlations could be calculated between the groups, gender, age and BMI. In this respect, a significantly increased oxidative stress was found in metalworkers, while parameters associated with physical inactivity and dietary habits were suspicious in office workers. Furthermore, the examination of both sexes is worth mentioning.

The weakness of this study is the limited number of participants, especially female subjects, which could, however, not be avoided since males dominated the workforce in the case of the examined metalworking enterprises. In addition, the lack of assessment of dietary habits is a shortcoming, and carbohydrate intake and total caloric intake could not be related to distinct groups or gender due to a lack of specific diets. Concerning the dietary habits of Austrians, an average daily energy intake of about 8500 KJ was reported by Farukuoye et al. [56], which was determined by two different questionnaires, i.e., food frequency questionnaires as well as a 7-day food record. The subjects usually had a mixed diet, as all kinds of special diets, such as vegetarian, vegan, etc., were an exclusion criterion, as was the use of food supplements and vitamins, alcohol consumption (more than “now and then”) and “smoking”. Nevertheless, definite differences in calorie intake and consumption occurred between metal and office workers, as might be indirectly assumed by several parameters from the present study. Another weakness of this study is the lack of a well-defined protocol of physical activity or the use of a pedometer. However, the subjects were comparable by the definition of moderate physical activity, i.e., two to three times sports per week, and thus differed only in exercise at work.

## 5. Conclusions

To summarize, there are significant differences in oxidative stress levels among metalworkers and office workers. Metalworkers indicated an excess risk of oxidative stress and inflammation-induced diseases, ranging from hypertension via cardiovascular diseases to cancer. Moreover, the oxidative stress profile of metalworkers was distinctly different compared to other heavy workers, suggesting an appropriate risk profile for each work area. The sedentary occupation in the case of office workers is associated with low physical activity. Therefore, they might be prone to diseases of affluence, e.g., disorders in glucose and lipid metabolism, which are also known to be associated with cardiovascular diseases or cancer. Thus, monitoring health conditions in both groups, e.g., during health check-ups, might preserve them from late damage. The implementation of oxidative stress biomarkers would be interesting from two points of view, i.e., on the one hand as a sensitive parameter to detect stress-related changes at an early stage, and on the other hand for long-term control and monitoring of appropriate therapy.

## Figures and Tables

**Table 1:** Baseline characteristics of different parameters in office employees and metalworkers.

Parameter	Office Employees	Metalworkers				
	(*n =* 29)	(*n =* 19)				
	Mean (95% CI)	Mean (95% CI)	*p* Group	*p* Sex	*p* Age	*p* BMI
Age, years	42.5 (39.9–45.0)	35.3 (32.1–38.5)	<0.001	0.008	-	-
Height, cm	178 (176–181)	177 (173–180)	0.439	<0.001	-	-
Weight, kg	80.1 (76.3–83.9)	78.3 (73.6–83.1)	0.569	<0.001	-	-
BMI, kg/m^2^	25.2 (23.9–26.4)	25.2 (23.6–26.7)	1.000	0.140	-	-
hsCRP, mg/L	0.99 (0.68–1.44)	1.75 (1.09–2.82)	0.080	0.251	0.135	0.385
DHEAS, µg/dL	263 (232–299)	343 (292–403)	0.017	0.040	<0.001	0.242
ACTH, pg/mL	19.6 (16.3–23.7)	31.22 (24.57–39.66)	0.005	0.421	0.889	0.290
TAC, mmol/L	1.81 (1.62–2.02)	1.72 (1.51–1.97)	0.614	0.724	0.800	0.799
EPA, U/L	8.49 (7.22–9.98)	7.03 (5.73–8.63)	0.188	0.359	0.719	0.723
Polyphenols, mmol/L	11.9 (11.7–12.1)	11.7 (11.5–12.0)	0.258	0.620	0.931	0.990
Uric acid, mg/dL	5.59 (5.28–5.90)	5.51 (5.11–5.90)	0.762	<0.001	0.673	0.068
TOC, µmol/L	141 (112–170)	225 (189–262)	0.001	0.189	0.205	0.218
Homocysteine, µmol/L	11.6 (10.7–12.7)	16.0 (14.3–17.8)	<0.001	0.004	0.463	0.623
LDL, mg/dL	120 (109–134)	129 (113–147)	0.460	0.224	0.137	0.077
HDL, mg/dL	57.6 (53.8–61.6)	55.6 (51.0–60.5)	0.550	0.830	0.938	0.058
LDL-HDL ratio	2.25 (1.95–2.54)	2.48 (2.10–2.85)	0.376	0.343	0.167	0.037
Cholesterol, mg/dL	199 (186–214)	214 (195–234)	0.253	0.121	0.102	0.160
Cholesterol/HDL ratio	3.45 (3.16–3.78)	3.84 (3.43–4.30)	0.170	0.190	0.210	0.013
Triglycerides, mg/dL	82.8 (71.4–96.0)	126 (105–153)	0.001	0.010	0.301	0.058
Glucose, mg/dL	90.2 (87.9–92.6)	87.9 (84.9–90.9)	0.256	0.115	0.422	0.229
HbA1c, mmol/mol	31.1 (30.2–31.9)	32.9 (31.8–34.0)	0.016	0.051	0.047	0.875
Copeptin, pmol/L	5.01 (3.82–6.56)	6.21 (4.43–8.69)	0.358	0.114	0.914	0.440
OL, mosmol/kg	285 (283–286)	289 (287–290)	<0.001	0.001	0.199	0.597
Creatinine, g/L	1.23 (1.04–1.45)	1.61 (1.26–2.06)	0.087	0.974	0.390	0.711
Adrenaline, µg/g	3.08 (2.41–3.93)	1.58 (1.10–2.27)	0.004	0.342	0.036	0.749
Noradrenaline, µg/g	12.9 (10.7–15.6)	14.6 (11.1–19.4)	0.487	0.446	0.478	0.991
Dopamine, µg/g	147 (126–170)	169 (135–212)	0.319	0.136	0.335	0.736
AMH, ng/mL	5.03 (4.00–6.07)	5.91 (4.61–7.22)	0.328	<0.001	0.022	0.994
Androstenedione, ng/mL	1.88 (1.64–2.15)	2.44 (2.05–2.89)	0.027	0.575	0.259	0.212
Cortisol, ng/mL	95.0 (84.9–106)	143 (124–165)	<0.001	0.067	0.515	0.067
FSH, mIU/mL	4.05 (3.07–5.33)	3.78 (2.67–5.36)	0.777	0.375	0.387	0.052
Free testo, pg/mL	7.75 (7.09–8.42)	8.02 (7.18–8.87)	0.643	<0.001	<0.001	0.392
Total testo, ng/mL	3.47 (3.07–3.87)	3.84 (3.34–4.35)	0.284	<0.001	0.004	0.148
HOMA-IR	0.96 (0.78–1.17)	1.51 (1.17–1.95)	0.011	0.464	0.838	0.002

BMI = body mass index; hsCR*P =* high sensitive C-reactive protein; DHEAS = dehydroepiandrosterone; ACTH = adrenocorticotropic hormone; TAC = total antioxidant capacity; EPA = endogenous peroxidase activity; TOC = total oxidant capacity; LDL = low-density lipoprotein; HDL = high-density lipoprotein; Glucose = fasting blood glucose; OL = osmolality; adrenaline, noradrenaline and dopamine µg/g creatinine; AMH= anti-Mueller hormone; FSH = follicle-stimulating hormone; free testo = free testosterone; total testo = total testosterone; HOMA-IR = homeostatic model assessment for insulin resistance.

**Table 2:** Comparison of oxidative and psychological stress, inflammation, and endocrinological parameter between males and females in office employees and metalworkers.

Parameter	Office Employees		Metalworkers	
	Males (*n =* 26)	Females (*n =* 4)	Males (*n =* 15)	Females (*n =* 5)
	Mean (95% CI)		Mean (95% CI)	
Age, years	43.4 (40.7–46.2)	37.9 (31.0–44.8)	36.9 (33.3–40.5)	27.7 (20.8–34.6)
Height, cm	181 (178–183)	165 (158–172)	179 (176–183)	163 (157–170)
Weight, kg	84.1 (80.1–88.1)	58.8 (48.7–68.78)	80.0 (74.8–85.2)	68.0 (58.0–78.0)
BMI, kg/m^2^	25.8 (24.5–27.2)	21.5 (18.2–24.7)	25.0 (23.3–26.7)	25.6 (22.3–28.9)
hsCRP, mg/L	0.91 (0.61–1.37)	1.56 (0.58–4.24)	1.65 (1.00–2.72)	2.41 (0.82–7.13)
DHEAS, µg/dL	274 (239–315)	224 (160–314)	365 (308–432)	252 (175–364)
ACTH, pg/mL	19.7 (16.1–24.1)	21.4 (13.0–35.2)	33.6 (26.1–43.1)	21.6 (12.6–37.0)
TAC, mmol/L	1.84 (1.64–2.07)	1.84 (1.64–2.07)	1.70 (1.48–1.96)	1.85 (1.36–2.51)
EPA, U/L	8.54 (7.17–10.18)	8.96 (5.98–13.44)	7.50 (6.06–9.27)	5.14 (3.29–8.02)
Polyphenols, mmol/L	11.9 (11.7–12.1)	11.8 (11.3–12.2)	11.7 (11.5–11.9)	12.1 (11.6–12.6)
Uric acid, mg/dL	5.76 (5.42–6.09)	4.87 (4.04–5.70)	5.75 (5.33–6.17)	4.26 (3.36–5.17)
TOC, µmol/L	134 (102–167)	174 (98.5–250)	218 (180–257)	260 (176–344)
Homocysteine, µmol/L	12.4 (11.3–13.5)	8.04 (6.45–10.02)	16.2 (14.5–18.1)	14.9 (11.8–19.0)
LDL, mg/dL	124 (111–139)	101 (76.5–133)	130 (113 -150)	122 (90.5–165)
HDL, mg/dL	57.3 (53.3–61.7)	59.0 (49.3–70.7)	55.6 (50.7–60.8)	55.6 (45.7–67.6)
LDL-HDL ratio	2.30 (1.98–2.63)	1.93 (1.13–2.72)	2.51 (2.11–2.92)	2.29 (1.42–3.16)
Cholesterol, mg/dL	203 (188–220)	179 (148–217)	218 (198–239)	194 (159–239)
Cholesterol/HDL ratio	3.54 (3.21–3.90)	3.04 (2.40–3.87)	3.91 (3.47–4.41)	3.53 (2.72–4.58)
Triglycerides, mg/dL	87.3 (74.4–103)	66.9 (45.1–99.1)	139 (114–169)	78.3 (51.1–120)
Glucose, mg/dL	90.8 (88.2–93.4)	87.5 (81.2–93.9)	88.7 (85.5–91.9)	83.8 (76.9–90.7)
HbA1c, mmol/mol	31.2 (30.4–32.2)	30.5 (28.4–32.7)	33.4 (32.2–34.6)	30.4 (28.2–32.8)
Copeptin, pmol/L	5.26 (3.93–7.05)	4.40 (2.16–8.95)	7.03 (4.93–10.02)	3.28 (1.52–7.05)
OL, mosmol/kg	285 (284–287)	282 (278–285)	290 (288–291)	284 (280–288)
Creatinine, g/L	1.22 (1.02–1.46)	1.32 (0.83–2.09)	1.64 (1.26–2.14)	1.44 (0.82–2.54)
Adrenaline, µg/g	3.30 (2.54–4.29)	1.93 (0.99–3.75)	1.55 (1.06–2.28)	1.74 (0.77–3.96)
Noradrenaline, µg/g	12.9 (10.6–15.8)	11.4 (6.9–18.9)	13.3 (9.9–17.8)	24.2 (13.0–45.1)
Dopamine, µg/g	140 (119–165)	189 (124–286)	163 (128–208)	202 (121–337)
AMH, ng/mL	4.53 (3.64–5.65)	0.67 (0.12–3.90)	6.20 (4.66–8.24)	1.96 (0.30–12.9)
Androstenedione, ng/mL	11.9 (1.63–2.13)	1.70 (0.76–3.79)	2.53 (2.07–3.10)	2.47 (1.41–4.31)
Cortisol, ng/mL	98.8 (87.9–111)	72.5 (47.2–112)	149 (127–175)	127 (71.5–224)
FSH, mIU/mL	4.63 (3.66–5.85)	3.29 (0.17–62.7)	4.07 (2.95–5.61)	3.35 (0.40–28.2)
Free testo, pg/mL	8.45 (7.62–9.36)	0.90 (0.44–1.84)	9.76 (8.47–11.3)	1.34 (0.67–2.67)
Total testo, ng/mL	3.45 (2.56–4.65)	0.12 (0.06–0.24)	4.77 (4.09–5.55)	0.19 (0.11–0.33)
HOMA-IR	1.00 (0.78–1.28)	0.80 (0.31–2.03)	1.61 (1.12–2.31)	0.96 (0.69–1.35)

BMI = body mass index; hsCR*P =* high sensitive C-reactive protein; DHEAS = dehydroepiandrosterone; ACTH = adrenocorticotropic hormone; TAC = total antioxidant capacity; EPA = endogenous peroxidase activity; TOC = total oxidant capacity; LDL = low-density lipoprotein; HDL = high-density lipoprotein; glucose = fasting blood glucose; OL = osmolality; adrenaline, noradrenaline and dopamine µg/g creatinine; AMH= anti-Mueller hormone; FSH = follicle-stimulating hormone; free testo = free testosterone; total testo = total testosterone; HOMA-IR = homeostatic model assessment for insulin resistance.

## Data Availability

The data presented in this study are available on request from the corresponding author.
